# Synergy of combined free nitrous acid and Fenton technology in enhancing anaerobic digestion of actual sewage waste activated sludge

**DOI:** 10.1038/s41598-020-62008-9

**Published:** 2020-03-19

**Authors:** Razieh Karimi, Seyed Mostafa Hallaji, Salar Siami, Ali Torabian, Behnoush Aminzadeh, Nicky Eshtiaghi, Soraya Zahedi

**Affiliations:** 1 Gorgan University of Agricultural Sciences & Natural Resources, Golestan, Iran; 2 Faculty of Engineering, Department of Civil Engineering, Monash University, Melbourne, Australia; 3 School of Environment, College of Engineering, University of Tehran, Tehran, Iran; 4 School of Engineering, Chemical and Environmental Engineering, RMIT University, Melbourne, Australia; 5 Catalan Institute for Water Research (ICRA), Girona, Spain

**Keywords:** Chemical engineering, Civil engineering, Bioenergy

## Abstract

In this study, actual swage waste activated sludge in batch reactors was employed to assess the synergistic effect of free nitrous acid and Fenton pre-treatments on enhancing methane production in the anaerobic digestion process. In addition to methane enhancement, the mechanisms driving the enhancement were also investigated via measuring enzymes activity and solubilisation of organic matter. This study revealed that the combined pre-treatments solubilised organic matter significantly more than the bioreactors pre-treated with individual FNA and Fenton. For understanding the influence of pre-treatments on solubilisation of organic matter, soluble protein, soluble polysaccharide and soluble chemical oxygen demand (SCOD) were measured before and after the treatments and it was shown that they respectively increased by 973%, 33% and 353% after the treatments. Protease and cellulase activity, as the key constituents of the microbial community in activated sludge, decreased considerably after the combined pre-treatments 42% and 32% respectively, which resulted in considerable methane enhancement. The results corroborate the synergy of the combined FNA and Fenton pre-treatment in degrading the organic and microbial constituents in waste activated sludge, paving the way for the big-scale implementation of these technologies.

## Introduction

Sewage Sludge treatment is the most cost-intensive process in wastewater treatment plants, accounting for around 60% of total operating costs^1^. Anaerobic digestion of sludge is an environmental and economical friendly method for sludge management because not only it dispenses with aeration equipment and related costs, but it also produces bio-methane, from which renewable energy could be generated. In anaerobic digestion, organic matter of sludge is transformed into CH_4_, CO_2_ and N_2_O^2^. In total, gasses produced in sludge treatment and disposal processes representent 40% of greenhouse gas emissions in wastewater treatment plants^3,4^. Enhancing methane production in anaerobic digestion of waste activated sludge harnesses greenhouse gas emissions and converts them to renewable energy^5^. Enhancement of methane production also reduces the volume of sludge via degradation of higher organic matter, which leads to lower sludge disposal costs and higher quality sludge for land application^6^.

Anaerobic digestion of waste activated sludge is often restricted by poor biochemical methane potential, low biodegradability and slow fermentation process^7^. In order to address these issues, various strategies have been employed in recent studies. Sólyom *et al*.^8^ and Ennouri *et al*.^9^ assessed the effect of physical pre-treatments of sludge on anaerobic digestion receptively with an application of microwave and thermal pre-treatments. Hallaji *et al*.^10,11^ assessed the influence of chemical, physical and physiochemical pre-treatment of sludge on methane enhancement and organic matter degradation in anaerobic digestion. Recently, Svensson *et al*.^12^ and Campo *et al*.^13^ also propounded methods of post and intermediate treatments of sludge with the intent of enhancing anaerobic digestion of sludge, dewaterability and quality of sludge. These treatments disrupt cell walls and extracellular polymeric substances (EPS), which improve the solubilisation of organic matter and methane production accordingly^14^.

As an environmentally friendly and economically favourable substance, free nitrous acid (HNO_2_) can be produced by nitration of anaerobic digestion liquor^15^. It generates hydrogen peroxide (H_2_O_2_), peroxynitrite (ONOO^−^), nitrogen dioxide (^.^NO_2_), hydroxide ion (OH^−^) and nitric oxide (^.^NO) which have inhibitory impacts on key microorganisms in wastewater treatment plants at part per billion (ppb) levels^16^. Zahedi *et al*.^17^ demonstrated that free nitrous acid with a concentration of 2.49 mg HNO_2_/L can reduce the cells’ viability in waste activated sludge by 80% after 5 hours. They also showed that following the reduction of microbial viability in waste activated sludge, which provides more biodegradable organic matter in the anaerobic digestion process, methane production increased by 20%.

Fenton reagent is formed by a combination of H_2_O_2_ and Fe^+2^ in a constant ratio, which Fe^+2^ functions as a catalyst for generating highly oxidising radicals (^.^OH)^18^. Hydrogen peroxide can also be produced in wastewater treatment plants via bio-electrochemical process^19^, which leads Fenton reaction to be partly renewable in wastewater treatment plants. When it comes to oxidation-reduction potential, Fenton reagent produces stronger radicals (+2.33 V) than hydrogen peroxide alone (+1.36 V) and ozone (+2.07 V), and it disrupts cell walls and EPS in sludge^18^. Therefore, it has good potential for enhancing methane production and degradation of organic matter in anaerobic digestion of sludge^20^. Erden and Filibeli^21^ demonstrated that 4 g Fe^+2^/kg TS and 60 g H_2_O_2_/kg TS with 60 minutes exposure time increased methane production by 19.4% in anaerobic digestion of waste activated sludge.

Wang *et al*.^22^ showed that in low FNA concentrations (lower than 8 mg/L) there are still some FNA-tolerable cells that can achieve homeostasis after the treatment, which finally prevent the better performance of the FNA pre-treatment in methane enhancement. Therefore, in this paper, it is hypothesised that combination of FNA pre-treatment with a relatively strong oxidising agent (like Fenton reaction) can amend the pre-treatment performance in destroying cell walls and solubilising extra/intracellular contents. Furthermore, FNA and Fenton reaction can be both produced from wastewater treatment processes^15,19^. Therefore, the combined application of these two technologies is a potentially sustainable approach in enhancing methane and accordingly, renewable energy production in wastewater treatment plants. Following the propounded hypothesis, this study aimed for investigating the synergistic effect of combined FNA and Fenton pre-treatments on (1) methane production from anaerobic digestion of waste activated sludge, (2) solubilisation of organic matter (mechanisms responsible for methane improvement) and (3) destruction of organic matter during the digestion. For FNA pre-treatment, two concentrations of 1.5 and 2.5 mg HNO_2_/L at 5 hours exposure time were employed^17^. For Fenton pre-treatment, two concentrations of H_2_O_2_ (25 and 50 g H_2_O_2_/Kg TS) with a constant ratio of H_2_O_2_/Fe^+2^ equals to 1/0.067 at 1 hour exposure time were employed^21,23–25^. Combination of these conditions was also used for assessing the synergistic effects of FNA and Fenton. To our knowledge, this is the first study, investigating the synergistic effect of combined FNA and Fenton pre-treatment on anaerobic digestion of waste activated sludge with consideration of organic matter solubilisation and enzymes activity for finding the possible driving mechanisms on methane production improvement.

## Results

### Influences of Fenton and FNA on solubilisation and enzymes activity

**Figure 1:**
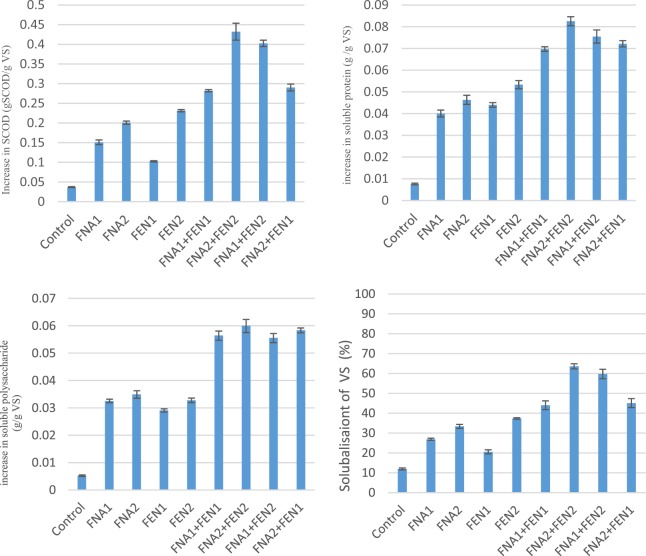
Biomass specific production of (1) SCOD, (2) soluble proteins, (3) soluble polysaccharides and (4) VSS after pre-treatment. Error bars represent standard error from triplicate measurements.

Figure 1 demonstrates the effect of Fenton and FNA pre-treatments on the solubilisation of organic matter. The amount of soluble oxygen demand (SCOD) in waste activated sludge samples before and after the treatments is shown in Fig. 1(1). According to the data shown, Fenton1 and Fenton2 pre-treatments increased SCOD by 0.10 and 0.23 g SCOD/g VS respectively, and FNA1 and FNA2 enhanced SCOD by 0.15 and 0.20 g SCOD/g VS respectively. However, significantly higher enhancement was achieved by combined FNA and Fenton pre-treatment in comparison to individual pre-treatments. Combined FNA2 and Fenton2 increased SCOD by 0.43 g SCOD/g VS, as the most effective pre-treatment in SCOD improvement among the presented pre-treatments.

The microorganisms existing in sludge are compounded mainly of protein (50%)^18^, so it is so important to assure that the organic matter solubilised by the pre-treatments is attributed to biodegradable organic matter such as soluble protein and polysaccharide. According to the measurements, soluble protein and soluble polysaccharides increased considerably in all pre-treated bioreactors (Fig. 1(2,3). The highest increase in soluble protein (0.082 g/g VS) and soluble polysaccharide (0.059 g/g VS) was obtained by FNA2 + Fenton2, which corroborate the synergistic effect of combined FNA and Fenton pre-treatments on disrupting microorganisms’ intracellular compounds and disintegrating organic matters than these pre-treatments alone. Individual FNA2 and Fenton2 pre-treatments increased soluble protein by 0.039 and 0.046 g/g VS and soluble polysaccharide by 0.029 and 0.032 g/g VS respectively.

Volatile suspended solids (VSS) is another indicator, reflecting the solubilisation of organic matter after pre-treatments. The amount of solubilisation of volatile solids (VS) was calculated, using $\frac{VS{S}_{in}-VS{S}_{out}}{VS{S}_{total}}\times 100$ formula. Figure 1(4) demonstrates the percentage of solubilised VS due to the pre-treatments. The amount of solubilised VS increased in the samples after pre-treatments, indicating that suspended solids transformed into soluble phase. Although the amount of increase was slight in the control reactor with 12%, the pre-treated reactors underwent a considerably high increase in solubilisation of the VS with up to 64% in combined FNA2 and Fenton2 pre-treatments.

Table 1 shows protease and cellulase activity, before (control reactor) and after pre-treatments. Enzyme activity is based on activity zone diameter (mm) in well agar diffusion method. The data is the average of triplicate tests with standard errors. Protease and cellulase activities reduced considerably after pre-treatments. Combined FNA2 + FEN2 reduced protease and cellulase activity by 42% and 32% respectively. The other combined pre-treatment (FNA1 + FEN1) led to 32% and 27% reduction in protease and cellulase activity respectively. Among the individual pre-treatments, Fenton exhibited a stronger effect on the enzyme’s activity in comparison to FNA, which indicates the stronger antimicrobial properties of Fenton technology.

**Table 1:** Key enzymes activity in WAS before and after the pre-treatments.

Reactors	Control	FNA1	FEN1	FNA1+FEN1	FNA2+FEN2
Protease activity (mm)	32 ± 0.9	28 ± 0.7	24 ± 1.1	21.7 ± 0.8	18.5 ± 0.2
Cellulase activity (mm)	37 ± 1	33 ± 0.3	28.7 ± 0.5	27 ± 0.8	25 ± 0.4

### FNA and Fenton effect on biochemical methane production

**Figure 2:**
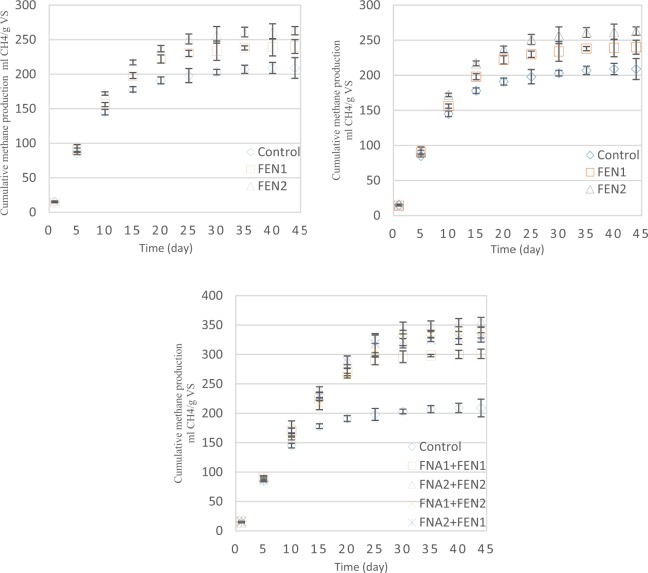
Cumulative methane generation from waste activated sludge with (1) FNA, (2) Fenton and (3) combined FNA and Fenton pre-treatments. Error bars represent standard error from triplicate tests.

Cumulative methane production from the bioreactors during the digestion process is shown in Fig. 2. According to the data shown, the highest increase in methane production was achieved from combined FNA2 + Fenton2 pre-treatments, accounting for 69%, followed by FNA2 + Fenton2 and FNA2 + Fenton1 with 61% and 57% respectively. However, methane production from individual pre-treatments did not transcend 26%, which was obtained from Fenton2 pre-treatment. FNA2 showed similar performance in comparison to Fenton2, which enhanced methane production by 25%.

**Figure 3:**
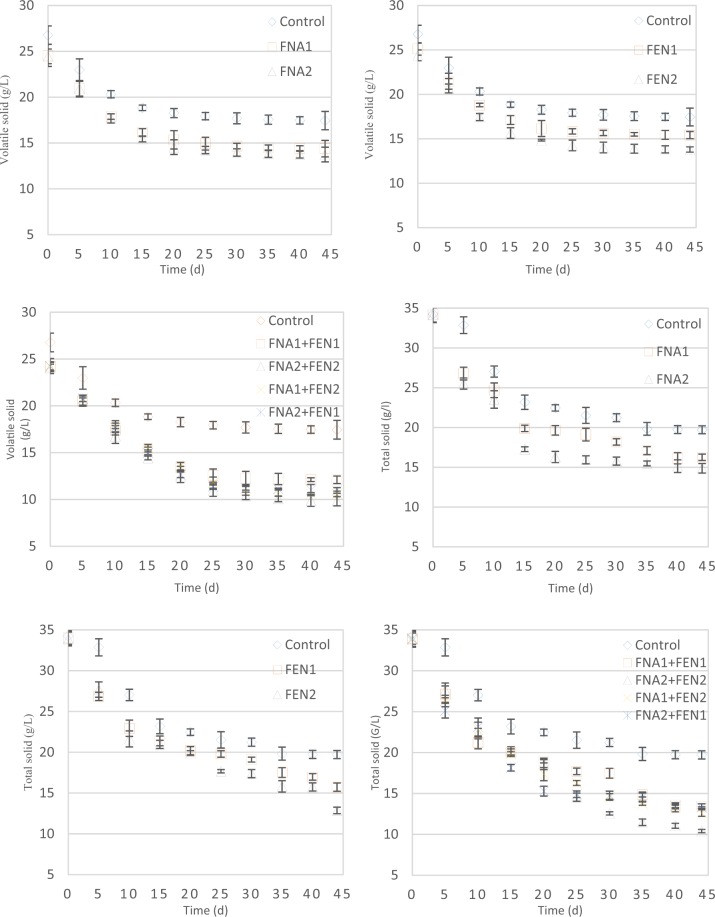
Sludge degradation during AD. Note: (1–3) demonstrate volatile solid degradation. (4–6) demonstrate total solid degradation. Error bars represent standard error from triplicate measurements.

### Degradation of organic matters

In this study, VS and total solids (TS) were measured regularly during the digestion process, and the trend of these phenomena is demonstrated in Fig. 3. During the digestion, FNA1 and FNA2 increased VS degradation by 6% and 8% respectively in comparison to the control bioreactor (Fig. 3(1). Fenton1 and Fenton2, also, increased VS degradation by 4% and 9% respectively (Fig. 3(2). The highest VS destruction obtained from pre-treated bioreactors with combined FNA2 + FEN2, accounting for 24% (Fig. 3(3). TS degradation experienced a similar pattern, in which combined FNA and FEN pre-treatments caused the highest degradation with up to 27% and individual FNA and FEN pre-treatments increased TS degradation by up to 14% and 20% respectively Fig. 3(4–6).

### Economic assessment

Economic assessment plays an important role in the feasibility of the pre-treatments. In this study, the and economic assessment was conducted using a desktop scaling-up study for the South Wastewater Treatment Plant of Tehran (additional data). According to the cost-benefit evaluations, it was shown that the net benefit obtained from the combined FNA2 + Fenton1 accounted for the highest amount with 460.12 €/d among the pre-treatments, followed by FNA2 + Fenton2 with 332.18 €/d. However, the lowest net benefits were achieved in Fenton2 pre-treatment with −678.67 €/d. Comparing FNA and Fenton pre-treatments, it was shown that FNA is considerably more cost-effective than Fenton pre-treatment. Furthermore, the combined FNA and Fenton pre-treatments accounted for the highest gross benefits, with a maximum of 2157.53 €/d achieved from combined FNA2 and Fenton2 pre-treatments. The lowest costs were also obtained in FNA pre-treatments, with a minimum of 319.10 €/d acquired from FNA1. It was also revealed that in FNA pre-treatments and Fenton pre-treatments, the price of FNA and H_2_O_2_ dominate the related costs with around 75% and 60% of the whole costs related to commercial chemicals.

## Discussion

Soluble organic matter in waste activated sludge samples increased in all pre-treated reactors significantly, which is attributable to the disruption of cell walls and EPS. Free radicals produced by FNA and Fenton reaction oxidise cell walls and EPS. The higher SCOD, soluble proteins, soluble polysaccharides and the higher solubilised volatile suspended solids (Fig. 1) after the treatments reflect the cell walls and EPS disruption. Wang *et al*.^25^ and Wei *et al*.^26^ also attributed the higher Soluble Kjeldahl Nitrogen and VS in sludge after FNA treatment to the changed floc structure of the sludge. Mechanisms of solubilising organic matter in Fenton treatment were also shown in Eqs. 1, 2 and 3^18^. In Fenton reaction, presence of ferrous iron accelerates the decomposition of hydrogen peroxide to hydroxyl radicals and hydroxyl anions in Eq. 1^27^. The hydroxyl radicals react with ferrous iron, which leads to Fe^+3^ and OH^−^ production (Eq. 2). There is a significant amount of organic matter (RH) in waste activated sludge. The hydroxyl radicals produced from Fenton reaction react with organic matter in sludge (RH), producing organic radicals (^•^R), which are highly reactive and capable of being further oxidised (Eq. 3)^20^.

1 $${{\rm{Fe}}}^{2+}+{{\rm{H}}}_{2}{{\rm{O}}}_{2}\to {{\rm{Fe}}}^{3+}+{}^{\cdot }\,{\rm{HO}}+{{\rm{OH}}}^{-}$$

2 $${}^{\cdot }{\rm{O}}{\rm{H}}+{{\rm{Fe}}}^{2+}\to {{\rm{Fe}}}^{3+}+{{\rm{OH}}}^{-}$$

3 $${\rm{RH}}+{}^{\cdot }{\rm{O}}{\rm{H}}\to {{\rm{H}}}_{2}{\rm{O}}+{}^{\cdot }{\rm{R}}$$

when it comes to the combined FNA and Fenton treatments, the combined mechanisms mentioned above can simultaneously improve the solubilisation process. Wang *et al*.^22^ illustrated that in low FNA concentrations (lower than 8 mg/l), some cells are still tolerable to FNA and by increasing the level of K^+^, Ca^2+^_,_ and H^+^ effluxes, they obtain ion homeostasis. Therefore, combining FNA with a strong oxidising reaction such as Fenton is likely to improve disruption of the FNA-tolerable cells in waste activated sludge.

The higher efficiency of combined pre-treatments in SCOD enhancement is attributable to the synergistic effect of radicals and oxidative chemicals released by FNA and Fenton reagent simultaneously. The SCOD enhancement from FNA pre-treatment, in this study, is in agreement with, but slightly higher than the research carried out by Zahedi *et al*.^17^ and Wang *et al*.^26^, which could be attributed to sludge specifications employed in this study.

The microorganisms existing in sludge are compounded mainly of protein (50%)^18^, so it is so important to rest assured that the organic matter solubilised by the pre-treatments is attributed to biodegradable organic matter such as soluble protein and polysaccharide. The results achieved in this study was slightly higher than the study carried out by Wang *et al*.^28^, in which combined FNA and thermal pre-treatments were applied to waste activated sludge and soluble protein, and soluble polysaccharide increased by up to 0.07 and 0.03 g/g VS respectively.

Protease and cellulase play a key role in the hydrolysis of organic matters and converting them to more readily biodegradable forms for anaerobic microorganisms’ consumption. Protease decomposes proteins to amino acids and cellulase catalysing the hydrolysis of polysaccharide to monoses^29^. Protease and cellulase activities were affected by the pre-treatments substantially. The higher level of pre-treatments resulted in the lower activity of the enzymes. This is especially evident in combined pre-treated reactors. The reduced activity can be attributed to antimicrobial properties of FNA and Fenton pre-treatments, which probably affect extracellular and intracellular constituents of the microorganisms. This effect can shorten the time needed for hydrolysis process in the AD process, which result in shorter hydraulic retention time (HRT) and smaller digesters that is of great significance from an economic and operational perspective.

The methane produced from the pre-treated bioreactors was considerably higher than that of the control bioreactor, which affirms the effectiveness of the employed pre-treatments in improving anaerobic digestion of waste activated sludge. Among the pre-treated bioreactors, those had been pre-treated with combined FNA and Fenton produced a significantly higher amount of methane, which corroborates the synergistic effect of these pre-treatments in enhancing methane production from anaerobic digestion of waste activated sludge.

Maximum methane production in pre-treated bioreactors was obtained in the fifth day of the digestion process. However, maximum methane production in the control bioreactor obtained in the second day of digestion. This difference can be attributed to overloading of the pre-treated bioreactors with soluble organic matter, which led to a delay of methane production^30^. Hallaji *et al*.^10^ revealed that using combined FNA and Fenton pre-treatment in anaerobic digestion of mixed primary and secondary sludge enhance methane production by up to 72%, which is relatively higher than that achieved in this study. However, methane enhancement obtained in this study was considerably higher than that achieved in Wang *et al*.^28^ with around 40%, in which they used combined FNA and thermal pre-treatments in anaerobic digestion of waste activated sludge. In Erden and Filibeli’s study^21^, the amount of methane enhancement from applying Fenton pre-treatments to waste activated sludge was 19.4%, which is relatively lower than that obtained in this investigation.

VS and TS disintegration are the most important indexes for organic matter destruction of sludge and anaerobic digestion efficiency^31^. As can be observed, the initial amount of VS and TS in pre-treated reactors is lesser than the control. This can be attributed to the effect of pre-treatments on declining VS and TS during the treatments’ exposure time. The increase in the level of pre-treatments resulted in VS and TS degradation. Fenton pre-treatment demonstrated a slightly stronger effect on VS and TS destruction than FNA in higher concentrations. Analogous patterns were achieved by Pilli *et al*.^32^ who pre-treated secondary sludge with Fenton reagent for enhancing anaerobic digestion.

Having taken all aspects into account, both FNA and Fenton improved methane production from waste activated sludge. This is mainly due to the release of more readily biodegradable organic matters into the soluble phase, caused by the pre-treatments (Fig. 1). Combined pre-treatments caused considerably higher methane production than FNA and Fenton pre-treatments individually, which is attributable to different radicals and oxidative chemicals, released by each of these pre-treatments. Methane production enhancement leads to higher bioenergy generation in wastewater treatment plants and lower greenhouse gas emission from sludge management process, which is of paramount importance from an environmental perspective. Furthermore, the improved TS and VS of the digested sludge pave the way for safer sludge reusing in farmlands and forests as an environmentally friendly and economically attractive technique.

The results from the economic assessments revealed that the pre-treatments would be considerably more economically favourable if FNA and H_2_O_2_ would be produced on-site in the wastewater treatment plant because the FNA and H_2_O_2_ considerably dominate the costs of the pre-treatments. In Fenton pre-treatment, particularly, this matter plays a vital role as the net benefit is negative. Using these technologies in the wastewater treatment plants with a lower pH of waste activated sludge can further improve the net benefit by reducing the acid costs as the second-highest cost in the pre-treatments.

In the future studies, a comprehensive economic assessment, in which on the one hand the costs associated to mixing and pumping of the pre-treated sludge, and on the other hand the economic and environmental benefits from higher sludge quality and lower methane emission should be conducted so as to comprehensively evaluate the feasibility of implementing these pre-treatments in full-scale.

## Conclusion

In summary, this study revealed that combined FNA and Fenton pre-treatments significantly enhance solubilisation of organic matter, microbial degradation, methane production, and organic degradation of waste activated sludge during anaerobic digestion, compared with these pre-treatments individually. Methane production enhanced by 69% in the bioreactors pre-treated with combined FNA2 + Fenton2, which is attributable to the synergistic effect of the pre-treatments and higher solubilisation of organic matter caused in these reactors. Importantly, in combined pre-treated reactors, soluble protein, as readily biodegradable organic matter, increased substantially and key enzyme’s activity reduced considerably, which corroborate the synergistic effect of the pre-treatments on disrupting cell walls and EPS in waste activated sludge. Besides, TS and VS degradation was enhanced by 26% and 24% respectively, compared with the control bioreactor at the end of the digestion process, which is of great importance from the operation and environmental perspective. This study revealed the potential of combined FNA and Fenton pre-treatments in improving anaerobic digestion of waste activated sludge.

## Methods

### Sludge characterisation

The thickened waste activated sludge was collected from belt thickeners in the South Wastewater Treatment Plant of Tehran. After collecting, the sludge was immediately transferred to the University’s laboratory and after measurements, it was kept at 4 °C and low pH. Characteristics of the waste activated sludge was as follows, which are achieved from triplicate tests with standard error: pH 6.45 ± 0.00, TS 40.10 ± 1.56 g/L, VS 32.00 ± 0.91 g/L, total suspended solids (TSS) 36.20 ± 1.03 g/L, volatile suspended solids (VSS) 29.40 ± 0.83 g/L, chemical oxygen demand (COD) 49.20 ± 1.12 g/L and SCOD 3.92 ± 0.10 g/L.

The inoculum used in this research for biochemical methane potential tests was harvested from the mesophilic digesters of the plant. Characteristics of the inoculum were as follows, which are obtained from triplicate measurements with standard error: pH 7.58 ± 0.00, TS 31.75 ± 0.93 g/L, VS 24.75 ± 0.86 g/L, TSS 27.75 ± 1.02 g/L, VSS 22.45 ± 0.79 g/L, TCOD 37.30 ± 0.63 g/L and SCOD 3.43 ± 0.15 g/L.

### Fenton and FNA methodology

For conducting FNA pre-treatment, first, the pH of waste activated sludge samples was reduced to 5.5 with 1 M HCl solution. Then, 4 M nitrite salt (NaNO_2_) was added to the mixtures, to provide the designated FNA concentrations in the sludge environment (Table 2). In the last stage, the mixtures were shacked with a shaker at 150 rpm for 5 hours. FNA concentration was computed by the equations N-HNO_2_ = (S_N-NO2_)/(Ka × 10 ^pH^) and Ka = e^−2300/(273+°C)^, in which °C is the temperature of the room (23 °C in this experiment), Ka is a constant which is dependent on the temperature and S_N-NO2_ is the nitrite salt concentration^33^.

**Table 2:** Pre-treatment conditions used in this research.

Pre-treatment	FNA (mg HNO_2_/L)	Fenton (mg H_2_O_2_/g TS)	Fe^+2^ (mg Fe^+2^/g TS)
Control	0	0	0
FNA1	1.5	0	0
FNA2	2.5	0	0
FEN1	0	25	1.68
FEN2	0	50	3.35
FNA1+FEN1	1.5	25	1.68
FNA2+FEN2	2.5	50	3.35
FNA2+FEN1	2.5	25	1.68
FNA1+FEN2	1.5	50	3.35

For conducting Fenton reaction, first, waste activated sludge samples were put into 1 L bottles, then their pH was decreased to 3 with H_2_SO_4_ solution (99%). In the next stage, iron salt (FeSO_4_) was added to the mixture, to produce the designated Fe^+2^ in the sludge environment (Table 2). Then, the designated hydrogen peroxide concentrations were added to the reaction. The ratio of H_2_O_2_/Fe^+2^ was set at 1/0.067 according to the literature^21,23–25^. The mixtures were finally shacked with a shaker at 150 rpm for 1 hour at ambient temperature so that the Fenton reaction would be approximately terminated^21,24^.

For combined FNA and Fenton pre-treatments, first, FNA pre-treatment was applied to waste activated sludge at 5 hours exposure time (pH = 5.5), then Fenton pre-treatment was applied at 1 hour (pH = 3). The combined conditions are shown in Table 2.

### Biochemical methane potential tests

**Figure 4:**
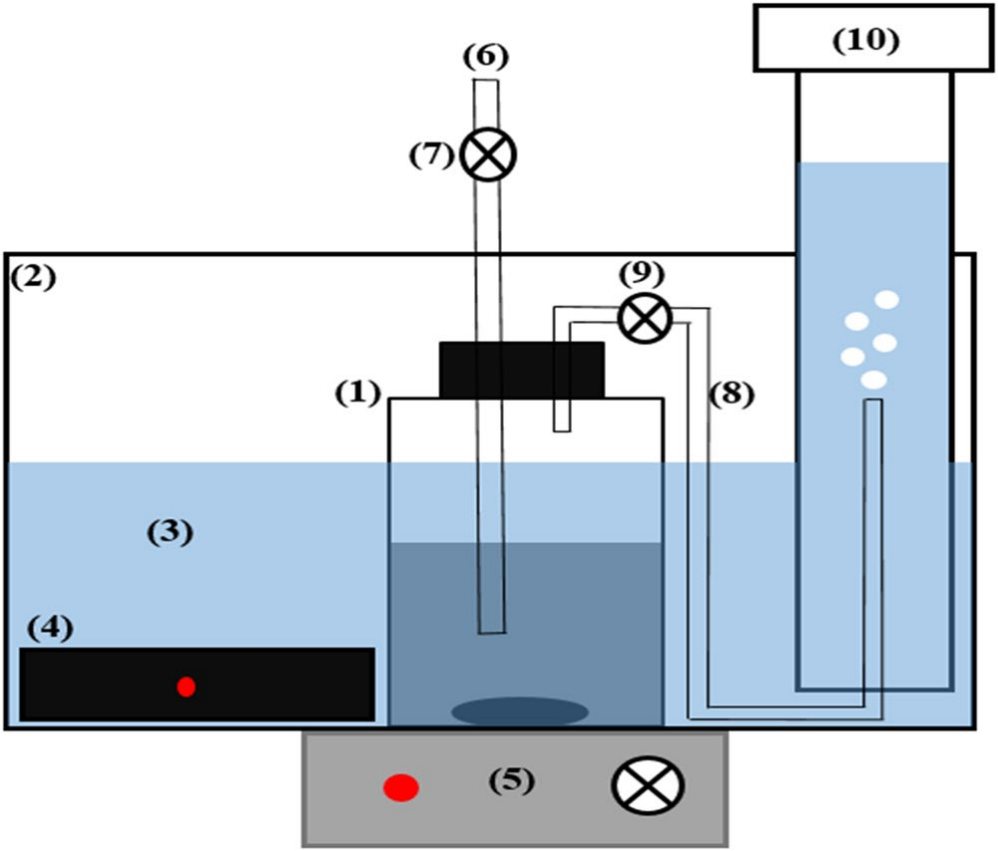
Schematic of the experimental system. (1) BMP reactor, (2) aquarium (3) saturated and acidified water, (4) automatic heater, (5) magnetic stirrer, (6) sampling pipe, (7) sampling control valve, (8) biogas collecting pipe, (9) biogas control valve, (10) graduated cylinder^10^.

In order to measure the volume of methane production from waste activated sludge, 27 batch reactors in addition to blank were carried out in 1000 mL glass bottles with a working volume of 500 mL (Supplementary Information). The schematic of the reactor is shown in Fig. 4. The ratio of inoculum and substrate (I/S) was adjusted to 2, based upon dry volatile solid^34^. The pH of treated waste activated sludge samples was adjusted to 7 and their temperature was increased to 37 °C prior to mixing with the inoculum, so as to prevent any temperature and pH shock to the inoculum. After mixing, the bottles were flushed with N_2_ gas for 1 minute (1 L/min), then they were put into the water bath, whose temperature was controlled at 36 ± 1 °C by automatic heaters. The bottles were permanently stirred by magnetic stirrers at 100 rpm, to provide adequate mixing and uniform temperature distribution. All tests were carried out in triplicate. The digestion process lasted for 44 days when biogas generation was approximately terminated.

### Economic assessment

The economic assessment was conducted using a desktop scaling-up study for the South Wastewater Treatment Plant of Tehran (Supplementary Information). The average flow of waste activated sludge entering anaerobic digesters is 575 m3/d, and the average biogas produced from 6 anaerobic digesters is 10800 m3/d. For the economic assessment of the employed pre-treatments, the costs and gross benefits were evaluated in the full-scale wastewater treatment plant, using commercial chemicals. In the economic assessment, the price of chemicals was considered as the “cost”, the price of electricity production from the improved methane was considered as the “gross benefit” and the difference between the cost and gross benefit was considered as the “net benefit”.

### Analytical methods

Routine experiments on sludge quality such as TS, TSS, VS, VSS, COD and SCOD were measured according to standard methods for the examination of water and wastewater^35^. For measuring soluble proteins before and after the treatments, Folin Phenol reagent was used according to Lowry’s method^36^. Soluble polysaccharides were also measured with phenol and sulfuric acid, according to Dubois’s method^37^. In order to separate soluble matter from suspended solids, 10 minutes centrifuge at 15000 rpm was first implemented, then the solution was passed through 0.45 μm pore size glass fibre filter, using Buchner funnel, and vacuum equipment.

The biogas volume was measured according to the liquid displacement method with an acidified liquid barrier (pH = 2) that was saturated with NaCl for minimizing the solubility of biogas^38^. Methane production was measured by gas chromatography (GC), using a Thermal Conductivity Detector (TCD). The temperature of the column and TCD were set respectively at 75 °C and 104 °C. In each measurement, 0.05 cc sample was injected to the equipment, and 1 minute exposure time was considered for each measurement. In this study, biogas and methane production were measured once daily and once every fifth day, respectively during the digestion process.

Key hydrolytic enzymes (protease and cellulase) activity was measured according to well agar diffusion method in our previous study^39–41^. For carrying out the enzymatic tests, methanogenic organisms should be first eliminated so as to decline potential errors^42^. For eliminating methanogens from the samples, heat treatment at 102 °C for 30 minutes and BESA (2-bromoethanesulfonic acid) were applied to the biochemical methane potential (BMP) reactors^43,44^. The samples then were maintained at 37 °C for 72 hours before being assessed for enzymatic activity.

## Data avalability

All data generated during this study are included in this published article afilend its supplementary information.

## Supplementary Information

Supplementary information.

Supplementary information2.
