# Supplementary material for: Synergy of combined free nitrous acid and Fenton technology in enhancing anaerobic digestion of actual sewage waste activated sludge
Source: Sci Rep. 2020 Mar 19;10:5027. doi: 10.1038/s41598-020-62008-9 (PMC7081239; doi:10.1038/s41598-020-62008-9)
Supplement: Supplementary file 2 — Supplementary information2. [file 41598_2020_62008_MOESM2_ESM.docx]

Synergy of combined free nitrous acid and Fenton technology in enhancing anaerobic digestion of actual sewage waste activated sludge

Razieh Karimi^1^, Seyed Mostafa Hallaji[^[[1]](#footnote-1)^](#corr)^,2,3^, Salar Siami^3^, Ali Torabian^3^, Behnoush Aminzadeh^3^, Nicky Eshtiaghi^4^ and Soraya Zahedi^5^

^1^Gorgan University of Agricultural Sciences & Natural Resources, Golestan, Iran

^2^Faculty of Engineering, Department of Civil Engineering, Monash University, Melbourne, Australia

^3^School of Environment, College of Engineering, University of Tehran, Tehran, Iran

^4^School of Engineering, Chemical and Environmental Engineering, RMIT University, Melbourne, Australia

^5^Catalan Institute for Water Research (ICRA), Girona, Spain

**Table S1. Soluble Chemical Oxygen Demand before and after pre-treatments (average of triplicate tests)**

| Reactors | SCOD before PTs (g/gVS) | SCOD after PTs (g/gVs) |
| --- | --- | --- |
| Control | 0.123±0.005 | 0.159±0.006 |
| FNA1 | 0.123±0.005 | 0.273±0.011 |
| FNA2 | 0.123±0.005 | 0.323±0.009 |
| FEN1 | 0.123±0.005 | 0.225±0.006 |
| FEN2 | 0.123±0.005 | 0.354±0.008 |
| FNA1+FEN1 | 0.123±0.005 | 0.405±0.008 |
| FNA2+FEN2 | 0.123±0.005 | 0.555±0.027 |
| FNA1+FEN2 | 0.123±0.005 | 0.525±0.013 |
| FNA2+FEN1 | 0.123±0.005 | 0.413±0.014 |

**Table S2. Soluble protein before and after pre-treatments (average of triplicate tests)**

| Reactors | Soluble Protein before PTs (g /gVS) | Soluble Protein After PTs (g/gVS) |
| --- | --- | --- |
| Control | 0.008±0.0003 | 0.016±0.001 |
| FNA1 | 0.008±0.0003 | 0.049±0.002 |
| FNA2 | 0.008±0.0003 | 0.055±0.002 |
| FEN1 | 0.008±0.0003 | 0.052±0.001 |
| FEN2 | 0.008±0.0003 | 0.062±0.002 |
| FNA1+FEN1 | 0.008±0.0003 | 0.078±0.001 |
| FNA2+FEN2 | 0.008±0.0003 | 0.091±0.002 |
| FNA1+FEN2 | 0.008±0.0003 | 0.084±0.003 |
| FNA2+FEN1 | 0.008±0.0003 | 0.081±0.002 |

**Table S3. Soluble polysaccharide before and after pre-treatments (average of triplicate tests):**

| Reactors | Soluble Polysaccharide before PTs (g / gVS) | Soluble Polysaccharide After PTs (g/gVS) |
| --- | --- | --- |
| Control | 0.183±0.005 | 0.188±0.011 |
| FNA1 | 0.183±0.005 | 0.215±0.038 |
| FNA2 | 0.183±0.005 | 0.218±0.040 |
| FEN1 | 0.183±0.005 | 0.212±0.035 |
| FEN2 | 0.183±0.005 | 0.215±0.038 |
| FNA1+FEN1 | 0.183±0.005 | 0.239±0.062 |
| FNA2+FEN2 | 0.183±0.005 | 0.243±0.065 |
| FNA1+FEN2 | 0.183±0.005 | 0.238±0.061 |
| FNA2+FEN1 | 0.183±0.005 | 0.241±0.064 |

**Table S4. Cumulative methane production (mL)** **during the digestion process (average of triplicate tests)**

| Day | 1 | 5 | 10 | 15 | 20 | 25 | 30 | 35 | 40 | 44 |
| --- | --- | --- | --- | --- | --- | --- | --- | --- | --- | --- |
| Control | 15±0.5 | 85±1.5 | 145±4 | 178±4 | 191±5 | 198±10.2 | 203±4 | 207±6 | 209±8 | 209±15 |
| FNA1 | 14±0.4 | 89±1.9 | 163±3 | 205±4.2 | 225±2.5 | 235±8.2 | 242±11 | 246±4 | 248±6 | 248±10 |
| FNA2 | 15±0.55 | 87±2.5 | 166±5 | 222±5.5 | 243±2.4 | 251±4.6 | 257±15 | 261±5 | 262±10 | 263±13 |
| FEN1 | 14±0.32 | 90±3 | 156±3 | 198±4.3 | 222±5.9 | 230±4.5 | 234±14 | 238±3 | 239±12.5 | 240±10 |
| FEN2 | 16±0.45 | 93±5 | 172±2.5 | 217±3.5 | 237±4.6 | 251±7.2 | 257±12 | 261±7 | 262±11 | 263±6 |
| FNA1+FEN1 | 16±0.44 | 91±3 | 170±4.6 | 224±2.9 | 270±6.1 | 289±6.4 | 296±10 | 298±2 | 300±7 | 301±8 |
| FNA2+FEN2 | 14±0.56 | 90±2.5 | 181±6 | 240±5 | 290±7.5 | 327±8.4 | 341±14 | 349±8 | 354±7 | 355±8 |
| FNA1+FEN2 | 15±0.6 | 90±1.5 | 158±3.4 | 216±10 | 264±4 | 308±10.5 | 328±13 | 334±5 | 337±10 | 337±9 |
| FNA2+FEN1 | 16±0.32 | 86±1.3 | 163±3.6 | 229±7 | 287±10.2 | 318±15 | 323±12 | 325±3 | 328±11 | 329±8 |

**Table S5. Total solids (g/l) during the digestion process (average of triplicate tests)**

| day | 0 | 5 | 10 | 15 | 20 | 25 | 30 | 35 | 40 | 44 |
| --- | --- | --- | --- | --- | --- | --- | --- | --- | --- | --- |
| Control | 34.3±1.02 | 32.9±1.05 | 27.0±0.7 | 23.2±0.9 | 22.5±0.4 | 21.5±1 | 21.2±0.5 | 19.8±0.8 | 19.7±0.5 | 19.7±0.5 |
| FNA1 | 34.2±1 | 26.8±0.8 | 24.7±0.92 | 19.9±0.4 | 19.6±0.6 | 19.1±0.8 | 18.2±0.4 | 17.1±0.5 | 16.1±0.7 | 16.3±0.4 |
| FNA2 | 34.2±0.9 | 25.5±0.7 | 23.5±1.1 | 17.3±0.3 | 16.3±0.7 | 15.9±0.5 | 15.8±0.5 | 15.5±0.3 | 15.1±0.8 | 14.9±0.6 |
| FEN1 | 34.0±0.89 | 26.8±0.5 | 22.9±1 | 21.4±0.6 | 20.2±0.5 | 19.8±0.4 | 19.1±0.3 | 17.5±0.6 | 17.0±0.4 | 15.7±0.5 |
| FEN2 | 33.9±0.85 | 27.7±0.94 | 21.6±0.98 | 21.0±0.5 | 19.9±0.3 | 17.7±0.2 | 17.4±0.5 | 15.8±0.7 | 15.7±0.5 | 12.9±0.4 |
| FNA1+FEN1 | 33.9±1 | 27.1±1 | 21.2±0.75 | 20.0±0.4 | 18.9±0.2 | 17.7±0.4 | 17.5±0.6 | 14.9±0.3 | 13.6±0.3 | 13.4±0.3 |
| FNA2+FEN2 | 33.8±0.82 | 27.6±0.9 | 22.3±0.64 | 20.2±0.5 | 18.6±0.7 | 14.5±0.5 | 12.6±0.2 | 11.5±0.4 | 11.0±0.3 | 10.4±0.2 |
| FNA1+FEN2 | 33.9±0.95 | 26.3±0.7 | 22.8±0.91 | 19.8±0.3 | 17.4±0.8 | 16.3±0.3 | 14.6±0.4 | 13.8±0.2 | 13.2±0.4 | 12.6±0.4 |
| FNA2+FEN1 | 33.9±1.02 | 25.1±0.9 | 23.1±1.1 | 18.1±0.4 | 15.3±0.6 | 14.9±0.4 | 14.8±0.5 | 14.6±0.5 | 13.4±0.3 | 13.3±0.1 |

**Table S6. volatile solids (g/l) during the digestion process (average of triplicate tests)**

| Day | 0 | 5 | 10 | 15 | 20 | 25 | 30 | 35 | 40 | 44 |
| --- | --- | --- | --- | --- | --- | --- | --- | --- | --- | --- |
| Control | 26.8±1 | 23.0±1.2 | 20.3±0.4 | 18.8±0.3 | 18.3±0.5 | 17.9±0.4 | 17.7±0.6 | 17.5±0.5 | 17.5±0.4 | 17.4±1 |
| FNA1 | 24.6±1.2 | 20.9±0.9 | 17.9±0.3 | 16.2±0.4 | 15.3±1 | 14.9±0.7 | 14.7±0.3 | 14.5±0.3 | 14.4±0.3 | 14.4±0.9 |
| FNA2 | 24.5±0.8 | 20.9±0.8 | 17.7±0.5 | 15.4±0.3 | 14.5±0.8 | 14.2±0.4 | 14.0±0.4 | 13.8±0.4 | 13.8±0.4 | 13.7±0.8 |
| FEN1 | 25.1±0.7 | 21.5±0.9 | 18.8±0.2 | 17.1±0.5 | 16.2±0.9 | 15.8±0.3 | 15.7±0.3 | 15.5±0.2 | 15.4±0.5 | 15.4±0.4 |
| FEN2 | 24.4±0.6 | 20.7±0.5 | 17.4±0.4 | 15.6±0.6 | 14.8±0.1 | 14.3±0.6 | 14.0±0.6 | 13.9±0.5 | 13.8±0.4 | 13.8±0.3 |
| FNA1+FEN1 | 24.3±0.8 | 20.6±0.5 | 17.4±0.5 | 15.2±0.4 | 13.3±0.2 | 12.6±0.7 | 12.3±0.7 | 12.2±0.6 | 12.1±0.2 | 12.1±0.4 |
| FNA2+FEN2 | 24.1±0.4 | 20.4±0.4 | 16.8±0.8 | 14.4±0.2 | 12.4±0.6 | 10.9±0.6 | 10.4±0.4 | 10.1±0.3 | 9.9±0.6 | 9.8±0.5 |
| FNA1+FEN2 | 24.2±0.5 | 20.6±0.6 | 17.8±0.6 | 15.5±0.4 | 13.6±0.4 | 11.8±0.6 | 11.0±0.5 | 10.7±0.4 | 10.6±0.1 | 10.6±0.3 |
| FNA2+FEN1 | 24.2±0.4 | 20.8±0.3 | 17.7±0.5 | 15.0±0.3 | 12.6±0.3 | 11.4±0.3 | 11.2±0.2 | 11.1±0.1 | 11.0±0.6 | 11.0±0.3 |

**Fig. S1. Bichemical methane potential system**

**
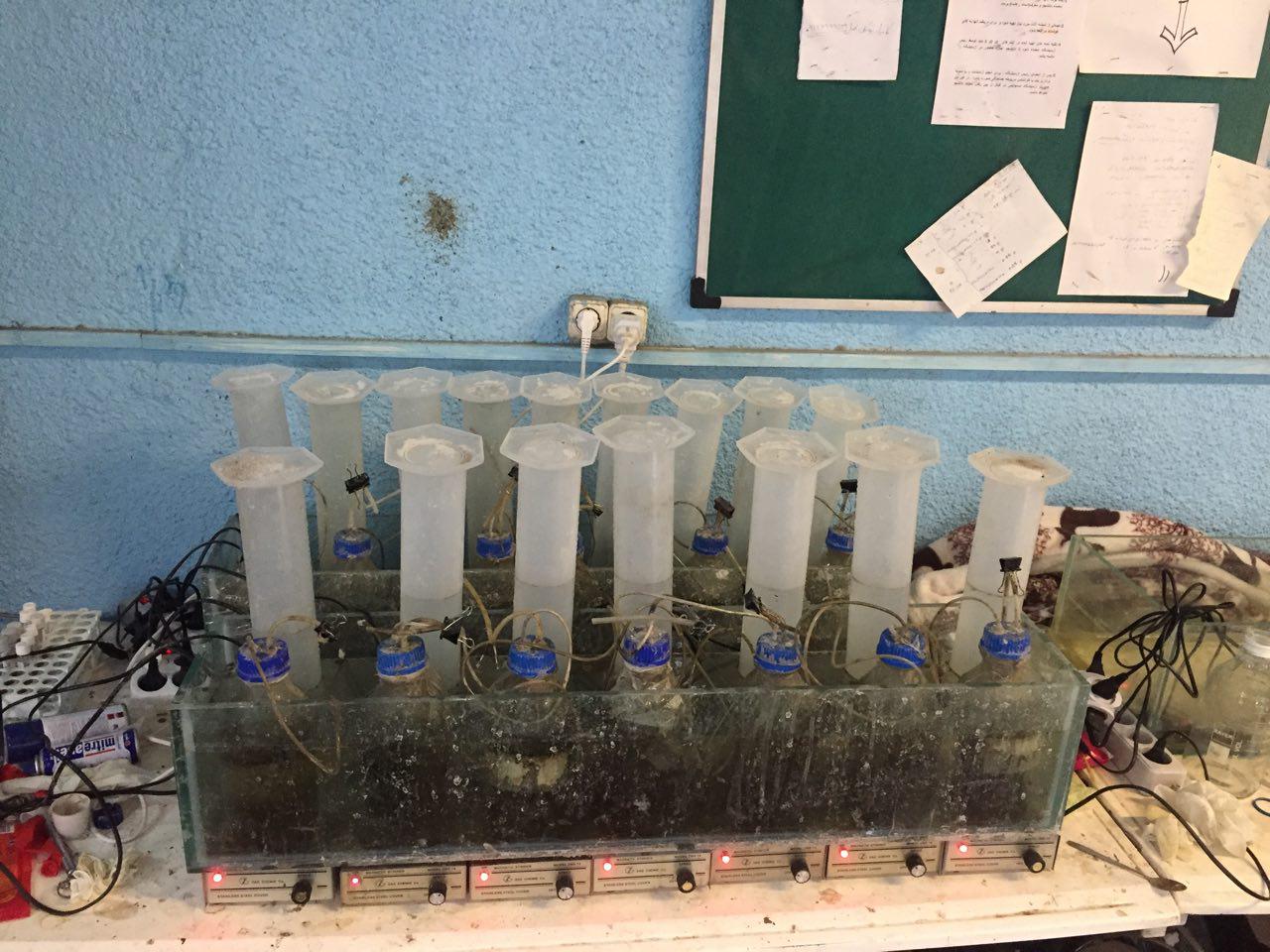
**

**Fig. S2. Soluble protein and polysaccharide measurements**

**
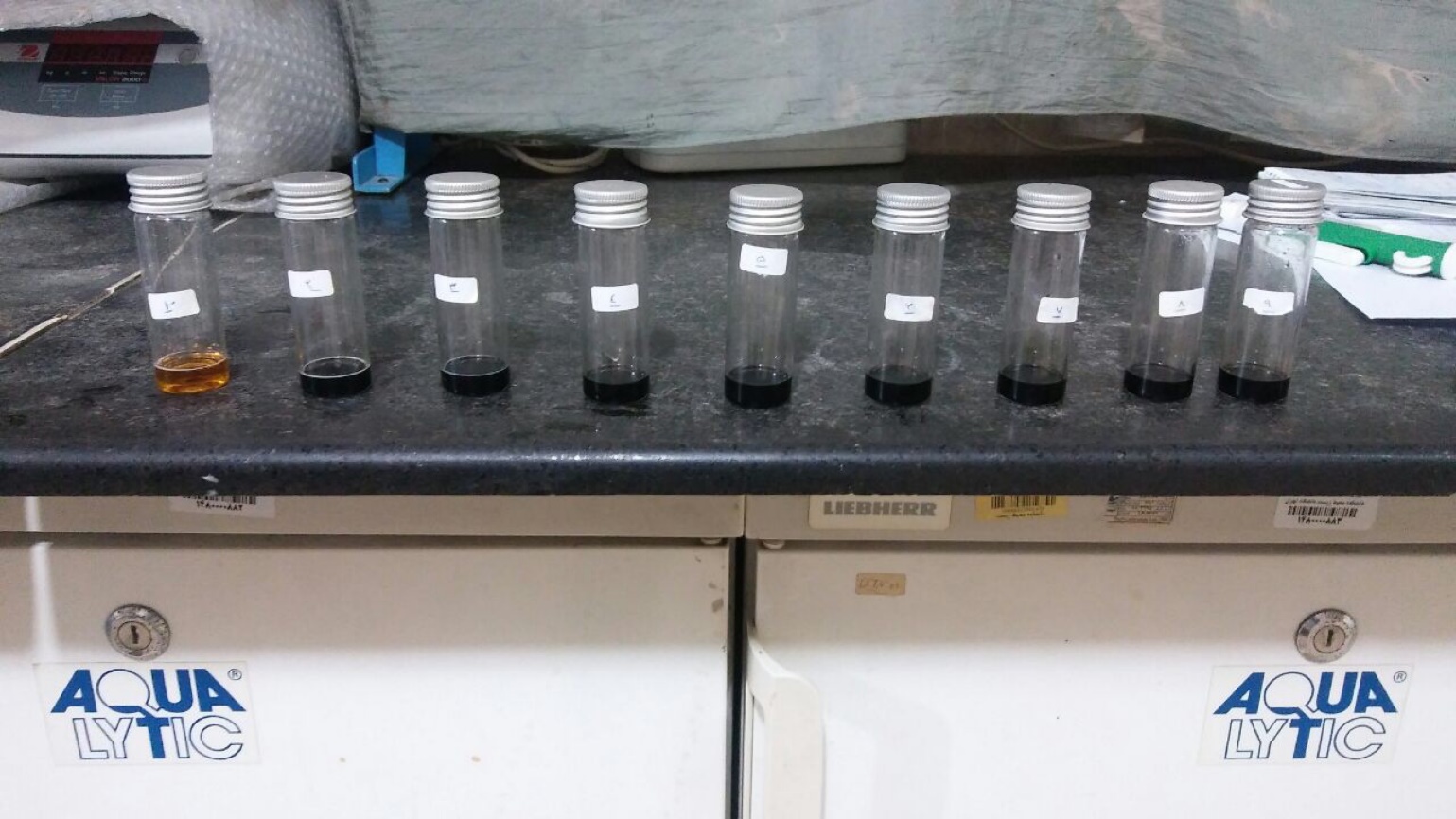
**

**
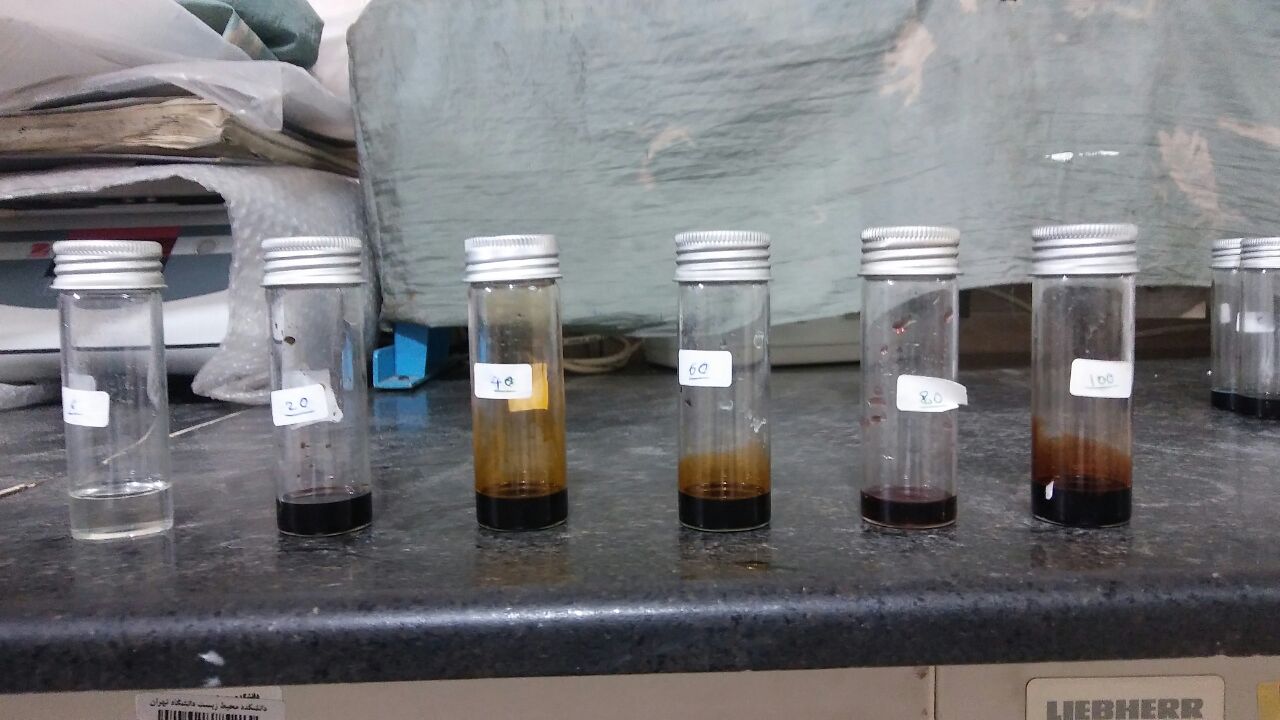
**

**Fig. S3 Gas chromatography analysis**

For measuring biogas contents, Gas Chromatography (GC) with Thermal Conductivity Detector (TCD) was employed. The temperatures of the column and TCD were set respectively at 75 ℃ and 104 ℃. In each measurement, 0.05 cc sample was injected to the equipment, and 1 minute exposure time was considered for each measurement. In the below figures, the biggest peaks illustrate methane content, which accounts for the highest biogas compounds. Followed by that CO_2_ accounts for the second-highest composition of biogas, shown by second highest peaks. The lowest peaks are attributed to N_2_ as a minor proportion of biogas.


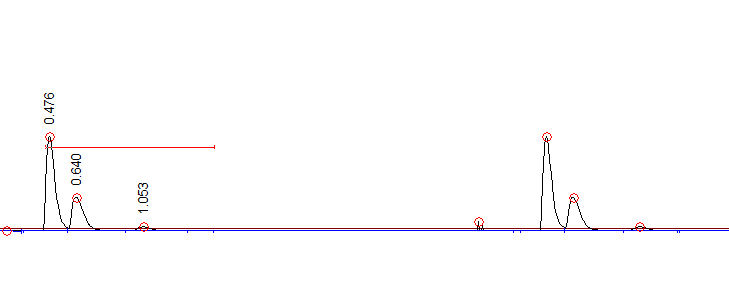

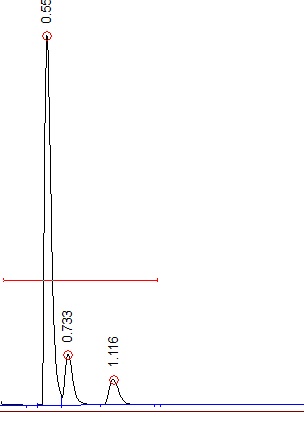

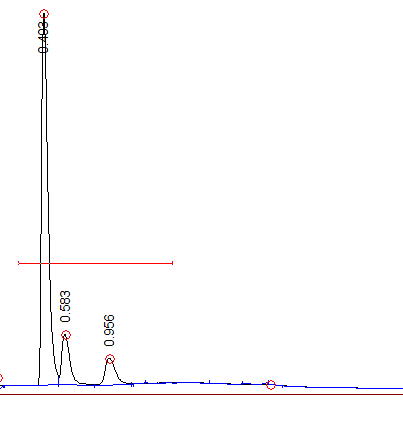

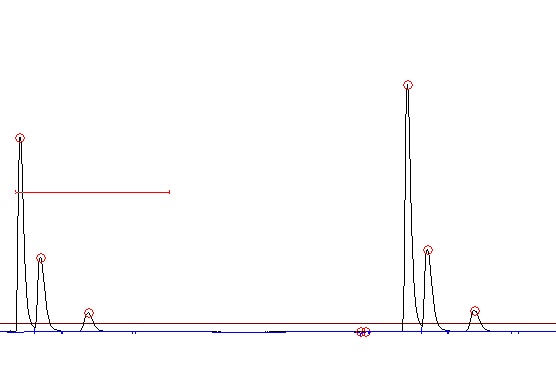

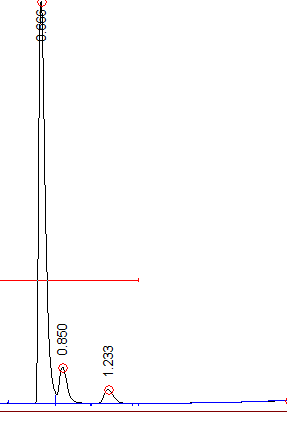

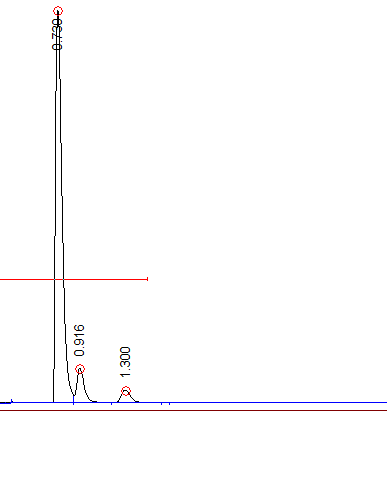


1. Corresponding author

   Email address: [mostafa.hallaji@monash.edu](mailto:mostafa.hallaji@monash.edu).

   Postal Address: Building 70, 21 Alliance Lane, Monash University, Clayton, 3800, Vic, Australia [↑](#footnote-ref-1)
